# Applying normalized p-control charts for early detection of local COVID-19 outbreaks

**DOI:** 10.1371/journal.pone.0357183

**Published:** 2026-10-20

**Authors:** Yuval Foox, Dan Yamin, Hila Chalutz-BenGal, Irad Ben-Gal

**Affiliations:** 1 School of Industrial & Intelligent Systems Engineering, The Iby and Aladar Fleischman Faculty of Engineering, Tel Aviv University, Tel Aviv, Israel; 2 Department of Industrial & Information Systems Engineering, Faculty of Engineering, Bar-Ilan University, Ramat Gan, Israel; University of Hamburg: Universitat Hamburg, GERMANY

## Abstract

The COVID-19 pandemic underscored the urgent need for early detection of local outbreaks to enable better resource allocation and effective mitigation. Outbreak identification largely depended on local infection rates, which were highly variable and influenced by daily testing volumes. Effective pandemic management requires accurate, adaptive methods that reflect the dynamic and localized nature of these rates, ensuring timely and appropriate interventions. This study seeks to improve the early detection and localization of COVID-19 outbreaks through Statistical Process Control (SPC) and health data analytics. We propose an adaptive outbreak-detection framework that combines a normalized p-control chart with an Amplitude-based labeler (ABL). For each area and day, the monitored statistic is a standardized positivity-rate score, computed from the observed daily positivity rate, the number of tests performed that day, and an area-specific baseline positivity estimate derived from recent historical observations. This calibration is followed by Phase II sequential monitoring using fixed three-sigma limits on the normalized scale and Western Electric decision rules. The framework accommodates varying daily sample sizes, captures regional interconnectedness, and supports parameter tuning using a temporally ordered train/test design. Accounting for daily testing fluctuations and spatial variability, it enhances the accuracy of local outbreak detection. Compared with the benchmark outbreak-detection methods considered in this study, the proposed framework was evaluated on its ability to predict outbreak onset within the following three days, achieving overall accuracy of 0.81 and averaged precision and recall of 0.83 and 0.79, respectively. Performance was further evaluated using the F_β_-measure, enabling a flexible trade-off between precision and recall depending on the relative cost of errors. SPC-based methods like ours can significantly improve early outbreak detection and localization. In the post-vaccination era, when targeted responses are key to controlling localized spread while avoiding widespread restrictions, health data analytics offer a valuable tool for informed decision-making.

## Introduction

The outbreak of SARS-CoV-2 in late 2019 created an unprecedented challenge for global public health, triggering deep and far-reaching socio-economic and healthcare consequences. Governments deployed a spectrum of containment measures, from strict lockdowns to aggressive vaccination campaigns, but these actions yielded mixed results, with recurrent infection waves and substantial mortality [1,2].

Geographical and environmental heterogeneity further shaped transmission. Population density, socioeconomic status, and local patterns of human mobility emerged as key determinants of disease spread, underscoring the need for public-health strategies tailored to local conditions [3–5]. Rapid detection of emerging outbreaks is, therefore, essential: early warnings enable timely containment and reduce pressure on healthcare systems.

Statistical Process Control (SPC) has been extensively used for decades in industrial, financial, and service systems to monitor, control, and improve process performance through data-driven decision-making [6–8]. Its strength lies in detecting variations and enabling timely interventions to maintain quality and efficiency. In recent years, SPC has been increasingly applied in healthcare systems, where it offers a promising surveillance framework for monitoring disease trends, identifying outbreaks, and improving patient safety [9–15].

Beyond SPC, recent healthcare analytics studies have also explored data-driven methods for detecting abnormal patterns and supporting early clinical warning. For example, Li et al. [16] proposed an adaptive outlier-detection method based on iterative generation of minimum spanning trees and demonstrated its applicability to medical data. Liu et al. [17] developed a clinical sepsis-screening score using lymphocyte count, international normalized ratio, and procalcitonin, illustrating the broader use of quantitative indicators for early detection in healthcare. These studies highlight the growing role of automated detection and screening methods in medical decision support, although they do not address sequential outbreak surveillance or SPC-based monitoring.

This transition reflects the growing recognition of SPC’s potential to support real-time public health responses and optimize healthcare delivery. In the use case considered, control-chart tools can transform noisy daily case counts into interpretable signals for action [9–11]. For example, Cildoz et al. recently proposed a weighted cumulative-incidence index to trigger statistically grounded alerts for new pandemic waves [12].

A broader literature reinforces SPC’s potential. Inkelas et al. applied binomial *p*-charts to city-level data [13]; Adeoti and Adekeye introduced a gamma-CUSUM that outperformed a normal-assumption CUSUM for Nigerian mortality counts [14]; Waqas et al. showed that an optimized EWMA chart signaled U.S. death-wave onsets earlier than Shewhart charts [15]. Two recent reviews highlight the surge of SPC methods and emphasize the need for charts robust to non-stationary sample sizes and spatial heterogeneity [18,19]. Methodological advances continue, including copula-based CUSUMs [20], backward CUSUM tests [21], and a genetic-algorithm design of SVSSI-np attribute charts that jointly optimized economic and statistical performance [22].

Despite this rich body of work, many of the above charts either assume fixed daily test counts or aggregate data over areas such that neighborhood spill-over or local factors are obscured. To bridge this gap, we introduce a variance-normalized *p*-chart that recalibrates as testing intensity changes and shares information across adjacent statistical areas. Specifically, we (i) derive normalized control limits with an amplitude-based labeler, and characterize the false-alarm behavior under the binomial sampling model; (ii) show empirically that the chart improves early outbreak detection accuracy across 241 monitored areas derived from 1,642 Israeli statistical regions, in which the chart achieves an overall F1 score of 0.81, thus 8% above the next-best method and more than 20% higher than the average of all other baselines; and (iii) provide locality-specific thresholds that trigger earlier, evidence-based municipal interventions.

Our implementation combines the normalized *p*-chart with an amplitude-based labeler (ABL) that distinguishes meaningful shifts from random fluctuation and enables automated large-scale model training. By tuning ABL parameters to COVID-19 time-series volatility [23–25], we enhance trend detection and reduce false alarms. The resulting framework attributes control-chart signals to subgroups of varying sizes, corrects for test-volume variance, and mitigates both false positives and false negatives; advantages illustrated in S1 Fig. Automated trend tagging and adaptive sample-size adjustment together deliver a robust early-warning system for public-health officials.

While our method builds upon the p-chart framework, it extends the concept beyond its traditional industrial context. Classical p-charts are designed to monitor the proportion of items conforming to a specification, with binary outcomes and control limits that account for sample size. In contrast, our normalized p-chart generalizes this principle to healthcare surveillance, where daily test volumes and underlying infection probabilities vary dynamically. The normalization preserves the interpretability and visual clarity of standard p-charts while ensuring statistical validity under non-stationary sampling conditions.

## Materials and methods

### 2.1 Proposed method

We developed a data-driven surveillance framework for early detection of local COVID-19 outbreaks. The framework combines Statistical Process Control (SPC)-based monitoring with automated trend labeling (using an amplitude-based labeler, ABL) and parameter optimization. We leveraged anonymized health data and cell phone mobility data to accurately predict the onset of outbreaks at a regional level. Specifically, we use a normalized p-chart to monitor area-level test positivity, while adjusting for daily variation in testing volume, and it incorporates regional information to strengthen local outbreak detection. The framework is designed primarily for adaptive Phase II monitoring, following historical calibration of area-specific baseline quantities. A complete step-by-step description of the RNOP algorithm is provided in S1 Algorithm.

### 2.2 Application design schema

We implemented a variety of components and procedures to construct a statistical outbreak-detection mechanism. We utilized an end-to-end procedure that can be implemented in similar pandemic outbreaks.

Fig 1 outlines the method and shows four main modules in the framework:

**Fig 1:**
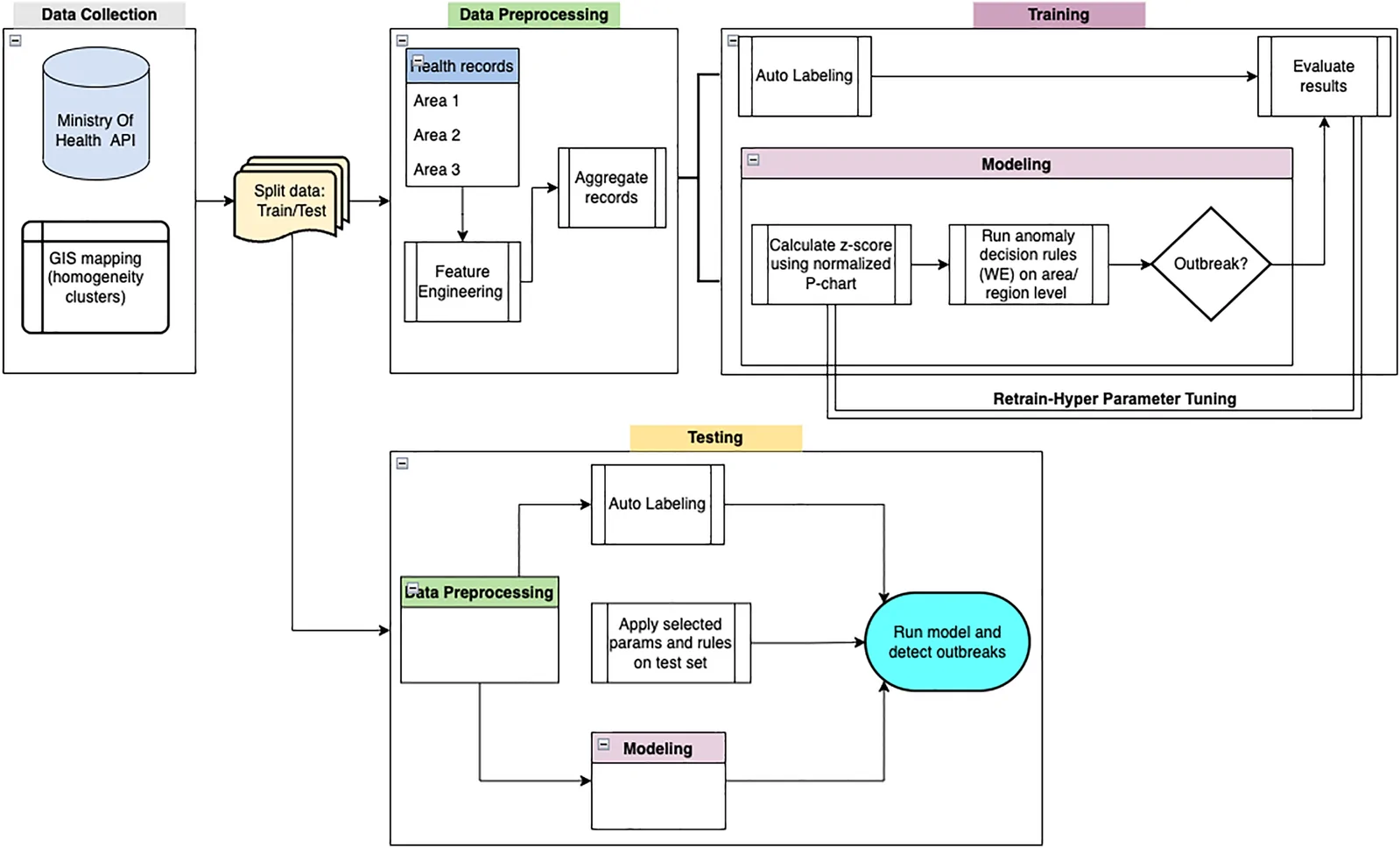
The proposed figure illustrates the comprehensive model design schema for early detection of local COVID-19 outbreaks (and potentially other outbreaks). The schema encompasses key modules: data collection, data preprocessing, model training (including auto-labeling, normalization, anomaly detection, model evaluation, and hyperparameter tuning), and model testing.

1. **Data Collection**: We imported raw data, which include: i) public COVID-19 data (taken in this use case from Israel’s Ministry of Health’s API [26]; and ii) public geographic and socio-demographic data, sourced from Yechezkel et al. [4].
2. **Data Preprocessing**: We created homogeneous clusters based on geographic location. We based the clustering on multiple numeric features (tests, cases, etc.), on-time aggregations (for example, sliding windows of 7/14 days), and *space* aggregations (e.g., area, region, neighborhood).
3. **Model Training**: This module included the following tasks:
  I. *Auto Labeling* – Before modeling, all data points were labeled automatically using trend detection (this part is further discussed below)
  II. *Normalization and Anomaly Detection* – normalized z-scores were calculated for each data point, while using normalized *p-chart*s and following conventional Western Electric (WE) rules to detect anomalies (aka outbreaks)
  III. *Model Evaluation* – the labeled data was used to train and evaluate the model
  IV. *Hyperparameter Tuning* – Using Grid Search optimization, we tuned multiple parameters (such as the WE rules, the labeling parameters, time and space aggregation parameters, etc.), and then validated the model performance in each area, based on the tuned parameters.
4. **Model Testing**: We replicated the training steps (except for hyperparameter tuning) and obtained a statistical testing result.

### 2.3 Data collection and splitting

We used publicly available daily COVID-19 health information (cases, tests, deaths, etc.) and socio-demographic data (age distribution, population figures, etc.) for Israel’s 1,642 statistical regions from 6 April 2020–15 April 2021 [4]. We mapped these 1,642 regions into 250 more homogeneous areas, neutralising the variance in small regions to ensure statistical reliability, as suggested in [4]. Health data were then apportioned to each sub-region in proportion to active cases sharing the same town and date. Finally, we divided the dataset into training and test sets, as common practice in machine-learning evaluation.

### 2.4 Exploratory data analysis (EDA) and feature engineering

Prior to model development, we examined the statistical properties of the regional COVID-19 time series to assess data adequacy and guide the monitoring design. The exploratory analysis revealed substantial day-to-day variability in testing volume, pronounced weekly seasonality, and unstable positivity estimates in areas with low testing activity. These observations motivated the use of temporal aggregation, area aggregation/exclusion criteria, and a normalized p-chart formulation that explicitly accounts for varying daily sample sizes.

For each area, we calculated the number of new cases, the number of new tests, and the daily test positivity rate. New cases were computed as the difference between consecutive cumulative case counts, while the positivity rate was calculated as the ratio of new cases to new tests (see Section 2.6).

Because conventional p-charts require adequate subgroup sizes for reliable estimation of control limits, we required a minimum of 30 daily tests and at least 25 valid observation days per area. Areas that did not satisfy these criteria were aggregated into larger neighboring areas or excluded from the analysis. After applying these data sufficiency requirements, 241 areas remained, representing approximately 93.5% of the Israeli population.

To characterize the temporal behavior of the data, we analyzed the national positivity rate, computed as the test-volume-weighted average across all monitored areas (374 daily observations). The positivity series exhibited multiple epidemic waves, substantial short-term autocorrelation, and a clear weekly testing cycle. The positivity-rate distribution was strongly right-skewed, while daily testing volumes varied by nearly two orders of magnitude across area-days, highlighting the need for variance normalization.

Stationarity was assessed using the Augmented Dickey–Fuller (ADF) and KPSS tests. The ADF test failed to reject the presence of a unit root in the raw positivity series (t = −2.25, p = 0.19) but rejected it after first differencing (t = −5.20, p < 0.001), indicating a non-stationary process with evolving baseline levels. Consistent with these findings, the autocorrelation function exhibited a slow decay pattern and significant dependence at short lags.

A Ljung–Box analysis identified statistically significant residual autocorrelation across multiple lags (all p < 10 ^−^ ^5^), with prominent peaks at seven-day intervals, confirming the existence of weekly seasonality and providing empirical support for the seven-day aggregation window described in Section 2.5. Furthermore, area-level trend analyses revealed substantial regional heterogeneity, with 154 of the 241 monitored areas (63.9%) exhibiting significant local trends. These findings support the use of adaptive baselines and area-specific monitoring within the proposed RNOP framework.

### 2.5 Records aggregation

In statistical control charts, it is a common practice to smooth variation due to cyclic behavior by either separating data lines within the chart or by subgrouping the data with a larger time unit (e.g., week rather than day). As an example, peak values often occur early in the week after the weekend, when more cases accumulate and people go to tests. Accordingly, to reduce weekly cyclic variation while preserving daily monitoring resolution, we considered 7-day and 14-day temporal aggregation windows. The 7-day moving average was selected as the default because it smooths weekday-related fluctuations while maintaining adequate sensitivity. To avoid carrying information from a previous state into a newly emerging trend, the moving-average window was reset after a detected trend change. Formally, for day t, the aggregation window was defined as $W_{t}=\{t-k+\text{1},\ldots ,t\},$ where $k=min(d_{t},U),$ and

$d_{t}$ is the number of days since the most recent trend change, and U=7is the default upper bound on the aggregation window.

### 2.6 Z-score calculation and detection rules

To accommodate variability in daily testing volumes across regions, we employed a normalized *p*-chart procedure. This approach standardizes positivity rates and stabilizes the false-alarm probability regardless of day-to-day fluctuations in sample size. The procedure consists of the following sequential steps:

#### 1) Compute daily positivity rate.

For each statistical area and day *i*, compute the daily test positivity proportion as:

$p_{i}$= number of positive tests on day *i*/ total number of tests on day *i*

2) Estimate the baseline (center line)

Calculate the average positive rate percentage:


$$\begin{array}{c} \hat{P}=\sum_{i}P_{i}/N\;\;, \end{array}$$

where N denotes the number of tested days (we used a sliding window of approximately 7 days)

3) Compute the standard deviation (*σ*)

${\hat{\sigma }}_{p}=\sqrt{\hat{P}\cdot (\text{1}-\hat{P})/n_{i}}$,

where $n_{i}$ denotes the number of tests on the *i*th day, thus reflecting the varying sample sizes. Under the null hypothesis of a stable underlying proportion, this statistic is approximately standard normal for $n_{i}$ ≥ 25.

4) Standardize (Z-score transformation)

Calculate the standardized statistic:


Zi=(Pi−P^)/σp^ =(Pi−P^)/ P^·(1−P^)/ni

#### 5) Define control limits.

Note that, on the normalized scale, (CL=0) and the nominal standard deviation is $\left(\sigma_{Z}=1\right)$; therefore, the three-sigma reference limits are (LCL=−3) and (UCL=+3).

Following conventional Shewhart chart practice, we set fixed three-sigma control limits, so that alarms are driven alarms indicate potential departures from the baseline. rather than by day-to-day changes in sample size:


$$\begin{array}{c} LCL=CL-\text{3}{\hat{\sigma }}_{p} \end{array}$$

$$\begin{array}{c} UCL=CL+\text{3}{\hat{\sigma }}_{p} \end{array}$$

In control-chart terminology, an *in-control* state represents stable conditions where variations are due to randomness alone. Under this assumption, each $Z_{i}$ follows an approximately standard normal distribution, and the probability of a false alarm (beyond ±3σ) is 0.27%. For independent standard-normal observations, considering only the single-point, two-sided three-sigma control-limit rule, this implies a nominal in-control average run length (ARL₀) of about 370 observations. This reference value does not describe the combined RNOP procedure. The framework was evaluated on its ability to predict whether an outbreak would begin within the following three days, as described in Section 2.8.

#### 6) Apply detection rules.

We apply two standard Western Electric (WE) rules to detect potential anomalies:

- Any single point exceeding/ falling ±3 for a normalized chart; or
- Two out of three consecutive points exceeding ±2 for a normalized chart, on one side of the center line.

These rules allow the system to identify both abrupt and sustained changes in outbreak trends [27].

Sections 7.1 and 7.2 in S1 Text provide additional details on how to estimate an in-control reference baseline for the positivity rate in each area based on historical observations.

#### 7) Implement nested regional alert logic.

We incorporated regional effects into the monitoring scheme in a nested manner. We generated an area-outbreak alert when an area-level rule met a threshold selected in hyper-parameter tuning (see “Hyper-parameter tuning”). Regional alerts were generated in a similar bottom-up approach when area-level rules were met. Proximity among regions was determined using Euclidean distance or origin–destination mobility matrices [4,5]. Fig 2 illustrates this nested approach, showing how alerting a region can influence the alert signal at the area level and vice versa.

**Fig 2:**
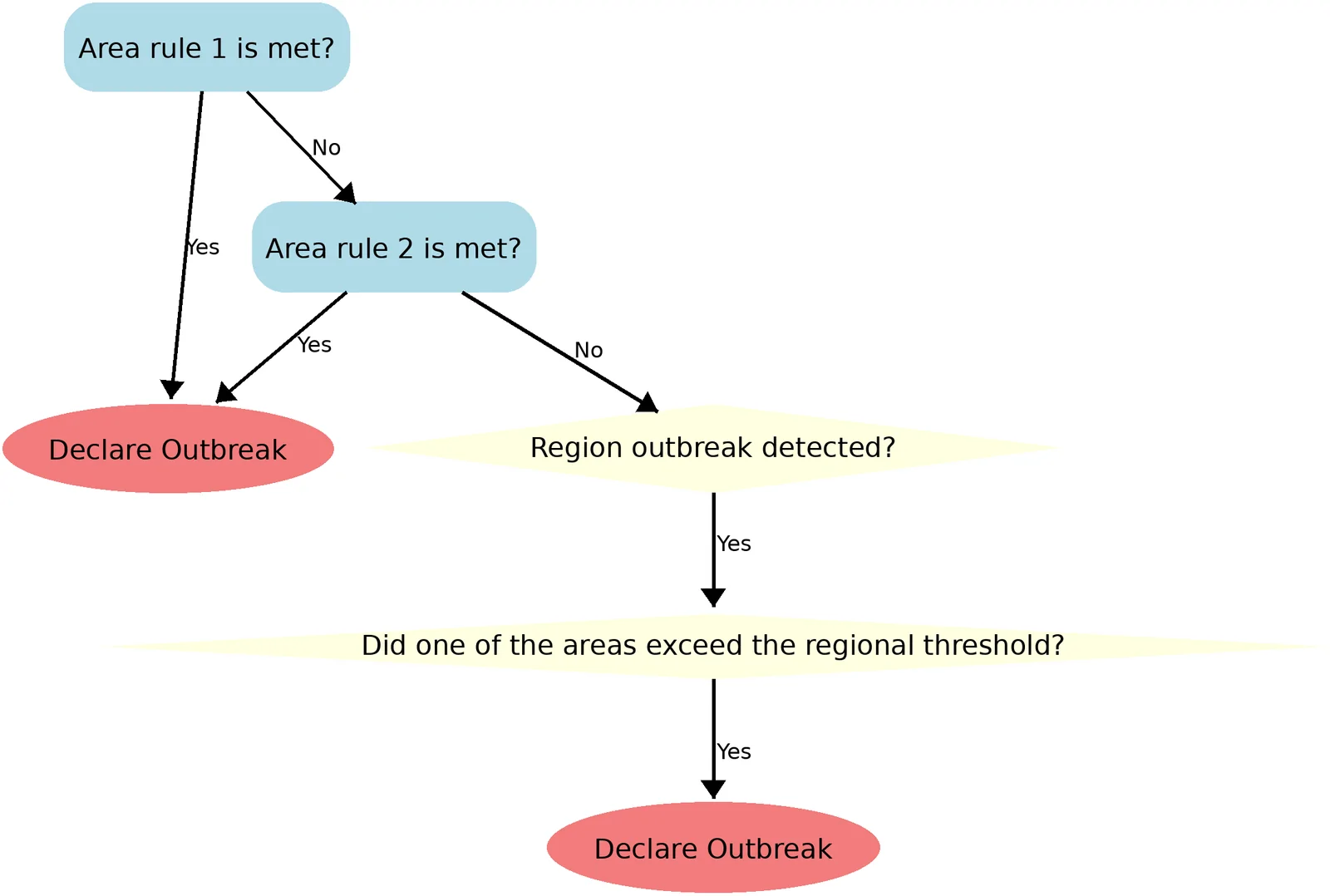
Regional nested effect diagram: if a region is alerted, it can impact the alert signal at the area level and vice versa. This nested information is captured and added to the model. The optimized region threshold is determined in the Hyperparameter tuning section. Note that proximity among regions can be determined with Euclidean distance measure or by origin-destination mobility matrices that capture the daily movements between regions [4,5]. Anomaly rules: (i) an anomaly point exceeding three standard deviations, and (ii) two out of three consecutive points exceeding two standard deviations.

This framework transforms noisy daily positivity rates into a statistically robust and interpretable control chart, maintaining consistent false-alarm behavior and improving the reliability of outbreak detection signals.

**Assumptions.** Independent daily counts within a region; binomial sampling; stable population denominators; baseline window reflects an in-control state.

**Limitations.** (i) Reporting delays can inflate $Z_{i}$; (ii) asymptomatic under-ascertainment may bias $\hat{P}$; (iii) the mobility matrix chosen to link regions influences nested-alert behaviour.

### 2.7 Automatic labeling/ tagging

We utilized the Amplitude Based Labeler (ABL) [23] to create labels for our model at scale, which were needed to learn and train our model. We used ABL, an automated tagging tool that efficiently labels data points in large-scale systems, surpassing manual tagging in accuracy, consistency, and cost-effectiveness. Unlike manual tagging, which can be subjective and time-consuming, ABL operates on predefined rules and guidelines, minimizing errors and ensuring consistent data labeling. ABL excels in trend detection within stochastic processes, striking a balance between recognizing significant yet minor trends and filtering out random fluctuations. The ABL component was used as an automated trend-labeling tool to support large-scale calibration and tuning of the monitoring framework. It is not itself the monitoring chart. Final evaluation of outbreak prediction performance was conducted against the operational outbreak definition described in Section 2.8.

To assess ABL’s effectiveness in our context, we performed a quantitative evaluation of its labeling accuracy. By comparing ABL’s outputs to a manually labeled subset of our data (used as ground truth), we found that ABL achieved a precision of 92%, a recall of 95%, and an F1-score of 93.48%. These metrics demonstrate the high reliability of our automated labeling approach. In a comparative analysis, ABL significantly outperformed the Ramer-Douglas-Peucker (RDP) method [24], which exhibited considerably lower accuracy. We determined the performance of ABL by two key parameters: the minimum trend (ideally a multiple of volatility or noise) and the maximum amount of noise allowed (the maximum period where no new high or low is achieved). By adjusting these parameters, we tailored ABL to match the specific COVID-19 time series data, enabling timely and accurate analysis of large-scale datasets in real-time scenarios. We further elaborated on the technical aspects of ABL and its implementation in S1 Text.

While ABL offers significant advantages, we acknowledge potential limitations, such as potential oversensitivity to short-term fluctuations in highly volatile periods.

Fig 3 shows how minimum trend sensitivity affects the labeler’s performance if it is tuned incorrectly. The left graph shows a greater degree of sensitivity than the right graph.

**Fig 3:**
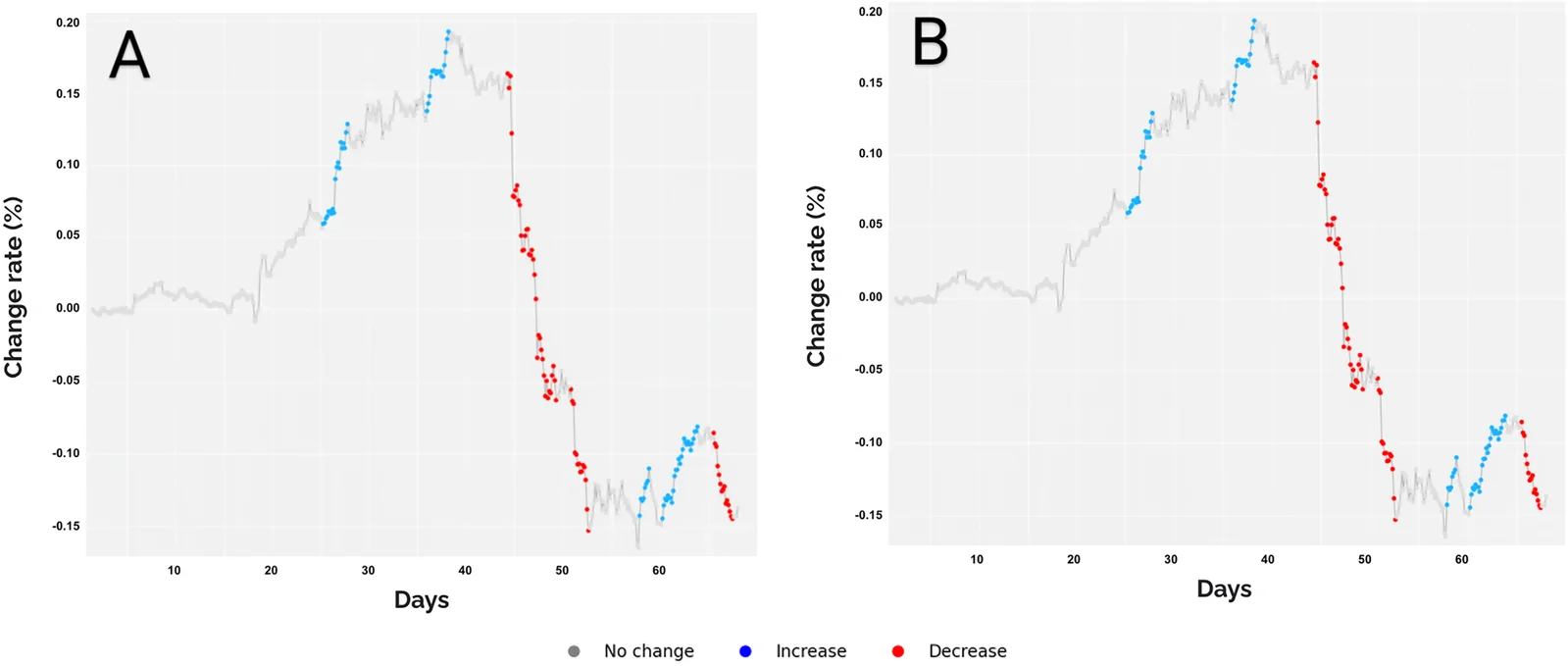
An illustrated example of the performance of the ABL method on generated data. Rate change (%) is represented by the Y axis, and the time (days) is represented by the X axis. Parameters: For the left graph (A) – maximum noise allowed = 15 days; Minimum trend = 7 days. For the right graph (B) – maximum noise allowed = 5 days; Minimum trend = 7 days. As seen from the minor differences, the ABL procedure is quite robust to those parameters.

After all procedures are complete and the parameters have been tuned, we refer to the resulting proposed approach as ‘*Regional Normalized Optimized p-Charting’* (RNOP).

### 2.8 Model evaluation and optimization

We evaluated the proposed control-chart scheme on a test dataset to assess its ability to predict whether an outbreak would begin within the following three days. Our performance metrics encompassed average Precision, Recall, FAR (False-Alarm Rate), and accuracy across all regional areas. To facilitate an objective evaluation, we formulated a specific, quantitative definition of an “outbreak” for this study: an outbreak is identified when the rate of increase in cases exceeds 25% compared to the mean of the previous 3 days.

To avoid ambiguity between automated trend labeling and the final evaluation target, we distinguish between two roles in the framework. ABL was used during model development as an auxiliary trend-labeling mechanism for large-scale tuning and calibration. Final outbreak prediction performance, however, was evaluated against the operational outbreak definition adopted for this study: an outbreak was defined as a case-growth rate exceeding 25% relative to the mean of the previous three days.

This definition was established through collaboration with Maccabi Healthcare Services (HMO), ensuring its relevance and applicability to real-world scenarios. By consistently applying this criterion across our dataset via the control-chart scheme, we maintained an objective and replicable method for outbreak identification.

Subsequently, we evaluated the model’s capacity to predict whether these operationally defined outbreaks would begin within the following three days. The model’s effectiveness was gauged using average precision, recall, and accuracy metrics for all regional areas, providing a thorough assessment of its predictive capabilities. This approach allowed us to quantitatively measure the model’s performance in identifying potential outbreaks before they fully manifest.

We evaluated six monitoring models: CUSUM, EWMA, *p*-chart, Hybrid Shewhart chart, Percentage Change Heuristic, and the proposed RNOP. The test criteria predicted whether an outbreak would start in the next three days.

To optimize RNOP, we implemented the F_β_-measure, blending precision and recall into a weighted harmonic mean. Adjusting the β parameter allowed us to favor precision (*β* < 1) or recall (β > 1) based on the scenario’s demands. We calibrated F_*β*_ to enable decision-makers to balance the tradeoff between missing an outbreak (Type II error) and false alarms (Type I error), which is crucial for optimizing resource allocation. Fig 6 visually represents this tradeoff by varying *β*, allowing informed choices based on priorities and available resources.

The F_*β*_ score is a valuable metric when both precision and recall should be considered, yet some tradeoff between the two is required, often based on economic and error-cost considerations. A higher F_β_ score indicates a better tradeoff between precision and recall. The choice of β reflects the relative importance assigned to precision and recall. A larger β places greater emphasis on recall when evaluating performance. Changing β alone does not alter predictions; it can affect the selected model configuration or decision threshold when used as an optimization criterion.

From a public health standpoint, achieving a balance between precision and recall is crucial. Precision is important to avoid wasting resources on false alerts, while high recall ensures that no actual outbreaks are overlooked, potentially preventing severe consequences. The F_β_ score provided us with a flexible tool, allowing us to prioritize precision or recall based on the evolving pandemic conditions, resource availability, and strategic objectives. This approach facilitated tailored decision-making, enabling authorities to respond effectively based on their specific needs and constraints.

### 2.9 Hyperparameter tuning

We employed a simple Grid Search to optimize the monitoring chart across all regional areas due to the relatively small number of model parameters and permutations. We considered the following parameter ranges in the Grid Search, determined in consultation with the HMO to account for practical considerations such as staff allocation, vaccinations, and supply chain tasks:

- WE rules: z-score threshold ranging from 1.5 to 3.5
- Time aggregation: 7 or 14 days
- Negative regional effect: 80–95% of the rule being met

To identify the optimal parameter set, we evaluated various combinations of parameter values using the Grid Search. For each combination, we calculated the average precision, recall, and accuracy metrics using a temporally ordered validation procedure within the training period. We present the results of this analysis in the subsequent section.

All analyses were executed in Python 3.11 using Pandas, Statsmodels, and SPC libraries; visualizations were produced with Matplotlib 3.8.

## Results

Six monitoring models were benchmarked: cumulative-sum (CUSUM), exponentially weighted moving average (EWMA), *p*-chart, the health-maintenance-organisation’s Percentage-Change Heuristic, the Hybrid Shewhart chart, and our Regional Normalised Optimised *p*-chart (RNOP). CUSUM is well-suited to sustained shifts, whereas EWMA is more sensitive to gradual historical changes [28]. The Hybrid Shewhart combines a *c*-chart for counts with an *I*-chart for individual observations to improve change-detection [29].

Table 1 compares Precision, Recall, F1, Accuracy and FAR (false-alarm rate). The false-alarm rate is the proportion of non-outbreak days on which a chart raises an alarm, calculated as FP/ (FP + TN). A lower FAR therefore means fewer unnecessary alerts and less alarm fatigue for decision-makers. Across CUSUM, EWMA, and the p-chart, overall F1 scores ranged from 0.62 to 0.68, and reported FARs ranged from 0.124 to 0.42. The Percentage-Change Heuristic attains high outbreak recall (0.89) but sacrifices precision, resulting in the highest FAR (0.745). The Hybrid Shewhart chart improves balance, delivering F1 = 0.75 with FAR = 0.21. RNOP achieves an overall F1 score of 0.81 and accuracy of 0.81, with a reported FAR of 0.176—approximately 16% lower than Hybrid Shewhart, 37% lower than the p-chart, and 58% lower than CUSUM. EWMA has a lower reported FAR (0.124), but lower outbreak recall (0.42 versus 0.76 for RNOP).

**Table 1:** Models’ performance.

Method	Outbreak			Not-Outbreak			All				FAR
	Precision	Recall	F1	Precision	Recall	F1	Precision	Recall	F1	Accuracy	
CuSum	0.67	0.73	0.7	0.67	0.6	0.63	0.67	0.66	0.66	0.67	0.42
EWMA	0.75	0.42	0.56	0.56	0.9	0.69	0.7	0.66	0.62	0.64	0.124
P-Chart	0.62	0.62	0.62	0.73	0.73	0.73	0.68	0.68	0.68	0.68	0.28
%-Change Heuristic	0.07	0.89	0.14	0.67	0.25	0.39	0.37	0.29	0.38	0.4	0.745
Hybrid Shewhart	0.69	0.65	0.67	0.82	0.8	0.81	0.76	0.73	0.75	0.735	0.21
Regional Normalised Optimised p-Chart	0.8	0.76	0.78	0.85	0.8	0.83	0.83	0.79	0.81	0.81	0.176

**Note:** Non-outbreak recall was averaged equally across areas, whereas FAR was calculated from pooled false-positive and true-negative counts across all areas. Consequently, the reported FAR is not necessarily equal to one minus the reported non-outbreak recall.

These results confirm that normalising for test-volume variance and incorporating regional information markedly boosts early-warning capability without a disproportionate increase in false alarms.

Fig 4 presents an intuitive visualization of the normalized p-chart applied to regional COVID-19 data. The chart plots standardized daily Z-scores with fixed three-sigma control limits (dashed lines at ±3). Signals are generated when a point exceeds these limits or when two of three consecutive points exceed ±2σ. This visual presentation demonstrates how the control limits and decision rules jointly identify meaningful upward shifts while filtering random fluctuations.

**Fig 4:**
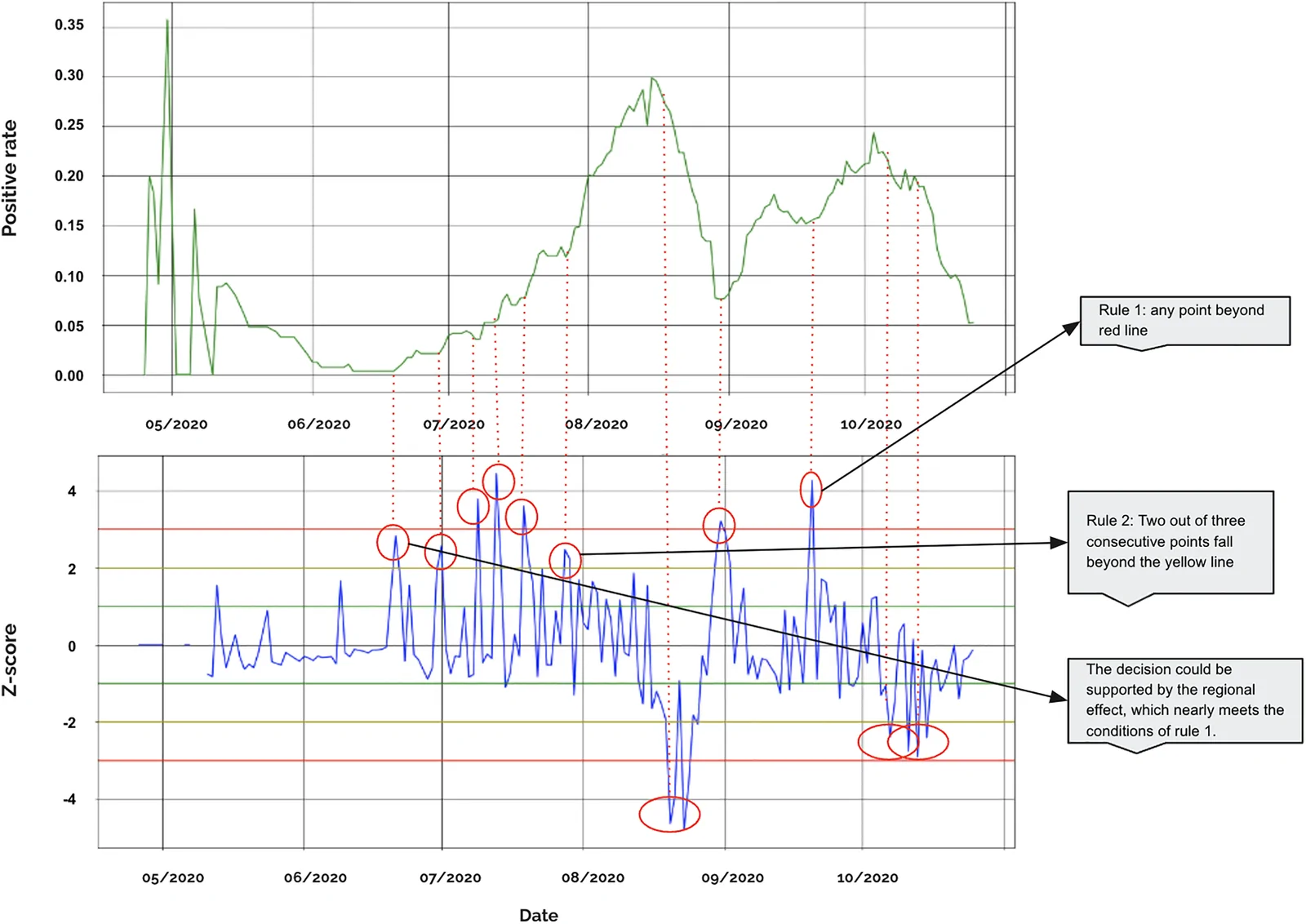
The Normalized *p*-chart example over real data between 05/2020 − 11/2020, location: Ramot Alon – Jerusalem.

The precision-recall curve in Fig 5 plots the trade-off between precision and recall for different thresholds. RNOP has the highest precision and recall, followed by the Hybrid Shewhart Chart. The *p*-chart and EWMA perform moderately, while the Percentage Change Heuristic and CuSum perform poorly.

**Fig 5:**
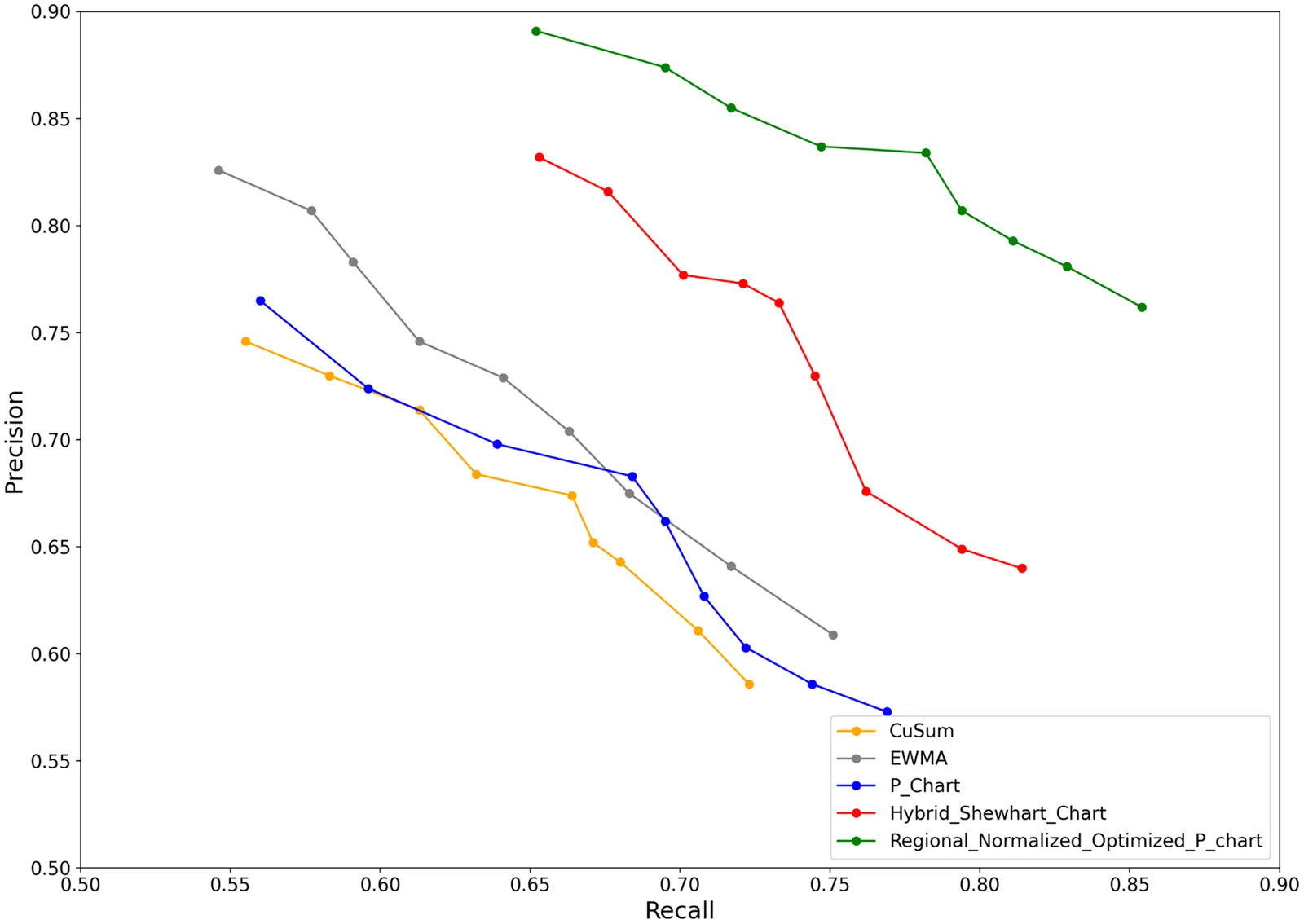
The displayed precision-recall curve evaluates the effectiveness of several outbreak detection methodologies, including CUSUM, EWMA, *p*-chart, Hybrid Shewhart Chart, and RNOP. The Percentage Change Heuristic method is excluded from this graph due to its significantly poorer performance. This curve plots recall on the x-axis against precision on the y-axis, demonstrating the balance between these two crucial metrics for each listed method.

To optimize RNOP, the F_β_-measure was implemented, blending precision and recall into a weighted harmonic mean. Adjusting the β parameter allows for favoring precision (β < 1) or recall (β > 1). F_β_ is useful when both precision and recall should be considered, yet some trade off is required based on economic and error-cost considerations. A higher F_β_ indicates a better tradeoff.

After our analysis, RNOP resulted in the highest accuracy and the highest F_β_ over most of the displayed β range, indicating its suitability for this scenario. This is due to its ability to handle trends, adjust to state changes, and not be affected by sample size.

Fig 6 visually represents how F_β_ scores change as the relative emphasis on precision and recall varies. RNOP performs best over most of the displayed β range; the Percentage Change Heuristic performs best at the largest β shown.

**Fig 6:**
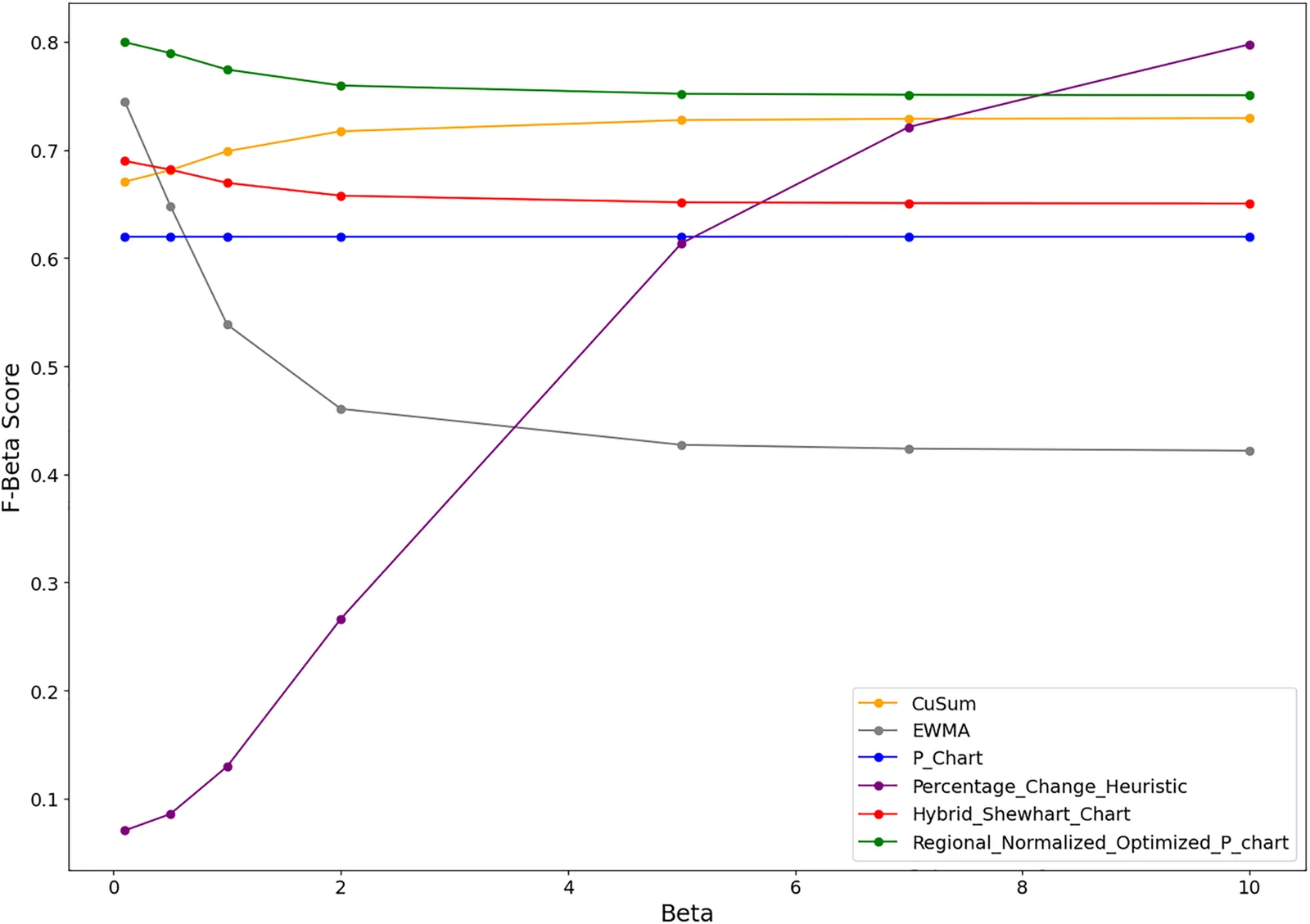
Comparison of the F_β_ for various trend detectors (models), while adjusting the Harmonic mean of the precision and recall. The x-axis shows the β value, starting with a higher weight on the precision while incrementally adding a higher weight to recall as the value increases. RNOP performs best over most of the displayed β range; the Percentage Change Heuristic performs best at the largest β shown. F_β_ scores are calculated from each method’s outbreak-class precision and recall at a fixed operating point.

Additional illustrative examples are provided in S1 and S2 Figs, which compare conventional varying-limit p-charts with our normalized version and show how the normalization improves interpretability and reduces false alarms.

## Discussion

In this study, we developed a simple yet robust method for early detection of COVID-19 outbreaks by combining normalized *p*-control charts with the Amplitude Based Labeler (ABL) algorithm. More broadly, the proposed framework should be interpreted as a hybrid surveillance system: an SPC-based sequential monitoring method whose calibration and tuning are supported by automated trend labeling, rather than as a purely classical fixed-limit p-chart applied in isolation.

Our framework was tested on data from over a thousand local outbreaks, and it demonstrated superior performance compared to other methods across multiple evaluation metrics. These results highlight the approach’s effectiveness in providing timely and accurate outbreak detection, which is crucial for implementing targeted interventions and optimizing resource allocation.

This work is motivated by the global challenge of COVID-19 containment. The worldwide spread of the disease has prompted nations to adopt a range of mitigation strategies, yet there is universal agreement on the importance of early pandemic detection to minimize impact and save lives [30]. Early identification of emerging infection clusters can significantly slow the spread of disease. Given that the pandemic’s impact often differs by region, a dynamic local-level surveillance system is essential to assess outbreak severity accurately and ensure that resources and social distancing measures are appropriately allocated. Nonetheless, achieving reliable monitoring and prediction of the virus’s spread presents significant challenges. One major issue is the stochastic nature of test positivity rates: substantial day-to-day variations in testing volume and other noise factors make trends difficult to interpret consistently. If these random fluctuations are ignored or misunderstood, decision-makers might draw erroneous conclusions and enact ineffective policies.

To address this challenge, our framework employs normalized p-control charts for outbreak monitoring. Although this approach differs from the classical industrial p-chart, it retains its core statistical rationale—tracking shifts in proportions over time—while extending the concept to domains characterized by variable denominators and stochastic fluctuations, such as daily epidemiological testing. These statistical process control charts enable public health authorities to identify meaningful deviations in disease trajectories in real time. When applied to relatively homogeneous regions, the charts effectively highlight significant increases in new cases, supporting early recognition of emerging outbreaks. Incorporating regional interdependencies further enhances contextual validity, ensuring that the resulting signals reflect true epidemiological dynamics and enabling more targeted and timely interventions.

Another key component of our approach is the ABL algorithm, which enhances trend detection amidst noisy data. ABL computes a dissimilarity score that quantifies the maximum deviation between the original data curve and a simplified version of that curve produced by the algorithm. It employs a sliding-window scheme to continuously track changes over time, dynamically focusing on the most recent data points. This design enables prompt recognition of shifts in the trend (such as sudden upticks in COVID-19 cases) while filtering out short-term random fluctuations. By tuning its parameters, ABL can be calibrated to detect meaningful trends within stochastic data, making it well-suited to track and predict disease spread during an outbreak.

A key strength of ABL lies in its efficiency and objectivity when tagging significant changes in large-scale data. Its automated nature eliminates the subjectivity and inconsistency that come with manual data analysis, ensuring a more uniform and error-reduced monitoring process. Furthermore, ABL operates in real time, which is invaluable for quickly monitoring, forecasting, and responding to a dynamic health crisis like COVID-19.

Despite these strengths, our approach has several limitations that suggest directions for future work. First, it relies on reported case numbers and test positivity rates; if a significant fraction of infections are asymptomatic or otherwise unreported, the data will under-represent the true disease prevalence. Consequently, predictions based solely on reported cases might be skewed and biased, underestimating the actual spread. Second, our framework assumes that each monitored region is relatively homogeneous. If there are large differences in population behavior or testing practices within a region, the performance of the *p*-control charts could be affected – potentially leading to missed signals or false alarms. Finally, while our method outperformed others in retrospective tests, it has not yet been fully validated in a real-time operational setting. Practical deployment may introduce challenges such as reporting delays or shifting outbreak dynamics that were not captured in our initial evaluation.

Addressing these limitations presents opportunities for future research. For instance, incorporating estimates of asymptomatic infections or adjusting for changes in testing rates could improve the accuracy of the control chart signals. Future work could also involve deploying this framework in live public health surveillance settings to evaluate and refine its real-time performance. Moreover, although our study focused on COVID-19, the general approach could be adapted to other infectious diseases or broader population health monitoring contexts. By extending the framework beyond the current pandemic and continuously improving its parameters, we can enhance the system’s robustness. For example, integrating additional data sources or machine learning techniques may further refine its performance. Another possible future extension, as suggested by an anonymous referee, is the expansion of the proposed approach to handle multivariate, adaptive control charts, and to incorporate extreme outliers and missing observations, which are beyond the scope of this study. Such enhancements would help healthcare organizations learn from data-driven insights and take appropriate actions more effectively, ultimately making health systems more resilient against both present and future outbreaks.

Although this study focuses on COVID-19 surveillance, the proposed framework is broadly applicable. The normalized *p*-chart can be adapted to monitor other public health indicators such as influenza outbreaks, hospital-acquired infection rates, or vaccination coverage [5]. Beyond healthcare, it may also be valuable in domains where proportions fluctuate with variable sample sizes, for example, manufacturing defect rates, web service error ratios, or quality-control processes in dynamic production environments. This generalization underscores the method’s flexibility and potential impact across diverse monitoring tasks and XAI applications [31].

In conclusion, our framework represents a significant advancement in pandemic response capabilities. It addresses the critical need for early detection and fine-grained monitoring of outbreaks, providing a robust data-driven strategy for outbreak management at the local level. While challenges like the under-detection of asymptomatic cases remain, these can be mitigated through ongoing model refinement and improved data collection. The adoption of normalized *p*-control charts combined with the ABL algorithm signals a shift toward more dynamic, evidence-based public health practices. This approach not only strengthens the ongoing fight against COVID-19 by enabling more timely and targeted interventions, but also lays the groundwork for stronger health surveillance systems in future pandemics. By focusing on real-time data and localized monitoring, our study illustrates a fundamental change in how public health emergencies can be understood and managed – ultimately optimizing resource distribution and facilitating timely interventions that safeguard lives and economies.

## Supporting information

S1 Fig A varying-limits p-chart (A) and a normalized p-chart (B). Both charts analyze the same data, while the normalized p-chart provides a clearer standardized representation of the shift beginning around day 13. Dashed horizontal lines indicate the ± 3σ control limits.
(PNG)

S2 Fig Conventional positivity-rate monitoring compared with the normalized p-chart. Variation in daily sample size can produce misleading raw positivity-rate signals; normalization accounts for sample-size-dependent variance and provides more reliable outbreak alerts.
(PNG)

S3 Fig National COVID-19 positivity rate over time. Daily positivity is shown together with the 7-day moving average across the study period.
(PNG)

S4 Fig Marginal distributions of daily positivity rate, daily confirmed cases, and daily tests across area-day observations. (PNG)

S5 Fig Autocorrelation function (ACF) and partial autocorrelation function (PACF) of the national daily positivity-rate series. (PNG)

S6 Fig Augmented Dickey-Fuller stationarity assessment of the raw national positivity-rate series and its first difference. (PNG)

S7 Fig Weekly seasonality and residual autocorrelation assessed using the ACF of the first-differenced series and Ljung-Box tests. (PNG)

S1 Text Supplementary methods describing Phase I baseline calibration, Phase II monitoring, automated trend labeling, exploratory analyses, and the full RNOP workflow. (DOCX)

S1 Algorithm Regional Normalised Optimised p-Chart (RNOP) pseudocode and evaluation procedure. (TXT)

S1 Code Jupyter notebook used to reproduce the extended exploratory data analysis and S3-S7 Figs. (IPYNB)
