# Supplementary material for: Applying normalized p-control charts for early detection of local COVID-19 outbreaks
Source: PLoS One. 2026 Oct 20;21(10):e0357183. doi: 10.1371/journal.pone.0357183 (PMC13682654; doi:10.1371/journal.pone.0357183)
Supplement: Supplementary file 1 [file pone.0357183.s008.docx]

# Supporting Information

## 7.1 Phase I calibration and baseline estimation

### For each area $a$, we estimated a local baseline positivity level using recent historical observations in the training period. Let $p_{a,t}$denote the observed positivity rate in area $a$on day $t$, and let $n_{a,t}$denote the number of tests performed on that day. The baseline positivity estimate $\hat{p}_{0,a,t}$was computed over a rolling historical window satisfying the minimum data sufficiency criteria described above. This estimate serves as the area-specific in-control reference level for subsequent monitoring. Because the epidemiological setting is nonstationary and testing intensity changes over time, the framework uses adaptive local baseline estimation rather than a single fixed baseline estimated once and held constant throughout the study period.

## 7.2 Phase II monitoring statistic and detection rules

### During monitoring, for each area $a$and day $t$, we computed the standardized positivity statistic

$Z_{a,t}=\frac{p_{a,t}-\hat{p}_{0,a,t}}{\sqrt{\hat{p}_{0,a,t}(1-\hat{p}_{0,a,t})/n_{a,t}}}.$

### Under the in-control assumption of binomial sampling around the local baseline, $Z_{a,t}$is approximately standard normal when the daily test count is sufficiently large. Monitoring was performed on this normalized scale using fixed three-sigma control limits ($\pm3$) and Western Electric rules. Specifically, an alert was generated when either: (i) a single point exceeded $\pm3$, or (ii) two of three consecutive points exceeded $\pm2$on the same side of the center line. This normalized formulation stabilizes the effect of varying daily sample sizes and improves interpretability relative to conventional varying-limit *p*-charts.

### An out-of-control signal is interpreted as indicating statistically unusual local positivity behavior relative to the recent baseline of that area. In the public-health context, such a signal can serve as an early warning indicator rather than a direct causal statement about underlying transmission mechanisms.

As seen in S1 Fig, the normalized *p*-charts can be easily interpreted and are clearer than the varying-limits *p*-charts.

S2 Fig illustrates the advantages of utilizing a normalized *p*-chart over the conventional positive rate calculation method. The normalized *p*-chart effectively differentiates between common and special causes of variation by considering sample size variations and calculating statistical control limits. In contrast, the conventional positive rate calculation needs to account for these factors, potentially leading to misinterpretation of the data and incorrect conclusions about the process's stability. The normalized *p*-chart provides a more accurate and reliable method for monitoring and analyzing the COVID-19 positivity rates, enabling better decision-making and outbreak detection.

## 7.3 Automatic Labeling / Tagging

Using automated metadata tagging is more efficient than manual tagging in large-scale systems. Manual tagging often negatively impacts data analysis since one person may subjectively categorize based on one rule, while another may categorize based on another. Compared to manual tagging, automated tagging follows specific guidelines, so error chances are greatly reduced. Furthermore, manual tagging requires a great deal of time, costs a lot, and is quite difficult to control in real time. An automated tagging tool, on the other hand, enables a framework for running and optimizing a machine learning layer over a "regular" SPC scheme. Detecting trends schemes have to deal with a built-in tradeoff of detecting important (but possibly small) trends while ignoring noisy trends arising from natural (random) variability in the considered stochastic process.

The Amplitude-Based Labeler (ABL) streamlines the tagging process in large-scale systems by automating the labeling of data points. This method surpasses manual tagging in efficiency, accuracy, and cost-effectiveness. ABL operates on predefined rules and guidelines, minimizing errors and ensuring consistent data labeling.

Additionally, ABL excels in trend detection within stochastic processes. It balances recognizing significant yet minor trends and filtering out random fluctuations, thereby extracting valuable insights without being misled by inconsequential variations.

Preliminary evaluations of the ABL method have shown its effectiveness. In a comparative study where 25 time series vectors were tagged both manually and with ABL, it demonstrated superior performance over other techniques, including the Ramer-Douglas-Peucker method [21].

Two parameters determine the performance of the ABL:

1. Minimum trend: This parameter should ideally be a multiple of the volatility or noise. In the context of COVID-19, this was defined as the minimum number of days to declare an outbreak.
2. The maximum amount of noise allowed: This is defined by the maximum period during which no new high (low) value is achieved. For the COVID-19 context, the maximum sensitivity or noise was defined as the maximum period where no new high (low) case count was achieved.

The ABL offers a robust and efficient approach to automating the data tagging process, enabling timely and accurate analysis of large-scale datasets in real-time scenarios. By setting these parameters, the ABL can be adjusted to match specific datasets, in this case, the COVID-19 time series data.

## 7.4 Exploratory Data Analysis Figures

The following figures present the results of the statistical time-series analyses described in Section 2.4. All analyses were conducted on the national daily positivity rate (test-volume-weighted mean across 241 areas, 374 observations). Code to reproduce all figures is provided in S1 Code (S1_code.ipynb).

## 7.5 Full Description of the RNOP Method

A complete formal specification of the Regional Normalised Optimised p-Chart (RNOP) framework is provided in S1 Algorithm. This file presents the full pseudocode required to reproduce the method from the supplied dataset.

Briefly, RNOP includes six stages: data ingestion and quality filtering; automated trend labelling using the Amplitude-Based Labeller (ABL); temporal aggregation and adaptive baseline estimation; normalised Z-score computation and control-limit application; nested regional alert propagation; and final hyperparameter optimisation and evaluation on the held-out test set.

Steps 0–4 correspond to the methodological components described in Sections 7.1–7.3, while Steps 5–6 specify the optimisation procedure, outbreak-label definition, and evaluation metrics used to obtain the results reported in Table 1.
